# Analysis of Morphological Changes in the Nucleus and Vacuoles of Acanthamoeba castellanii following Giant Virus Infection

**DOI:** 10.1128/spectrum.04182-22

**Published:** 2023-03-21

**Authors:** Sho Fukaya, Lisa Masuda, Masaharu Takemura

**Affiliations:** a Department of Applied Information Engineering, Faculty of Engineering, Suwa University of Science, Chino, Nagano, Japan; b Laboratory of Biology, Institute of Arts and Sciences, Tokyo University of Science, Shinjuku, Tokyo, Japan; c Laboratory of Biology, Graduate School of Mathematics and Science Education, Tokyo University of Science, Shinjuku, Tokyo, Japan; Centro de Investigacion y de Estudios Avanzados del Instituto Politecnico Nacional

**Keywords:** nucleocytoviricota, *Acanthamoeba*, giant virus, image analysis, medusavirus, nucleus, vacuoles

## Abstract

Acanthamoeba castellanii medusavirus is a member of the phylum *Nucleocytoviricota*, also known as giant viruses, and has a unique strategy of infecting Acanthamoeba castellanii and replicating viral genes in the host nucleus. Here, we show time series changes in the intracellular morphology, including the nucleus, of host cells infected with four types of giant viruses, including medusavirus, using time-lapse phase-contrast microscopy and image analysis. We updated our phase-contrast-based kinetic analysis algorithm for amoebae (PKA3) to use multiple microscopic images with different focus positions to allow a more detailed analysis of their intracellular structures. Image analysis using PKA3 revealed that as medusavirus infection progressed, the host nucleus increased in size and the number of vacuoles decreased. In addition, infected host cells are known to become smaller and rounder at later stages of infection, but here they were found to be larger than uninfected cells at earlier stages. These results suggested that the propagation mechanism of medusavirus includes the formation of empty virus particles in the host cytoplasm, packaging of the viral genome replicated in the host nucleus, and then the release of viral particles.

**IMPORTANCE** In this study, we quantitatively revealed how long the increase in host cell size or the increase in host nucleus size occurs after infection with giant viruses, especially medusavirus. To understand the underlying mechanism, we performed image analysis and determined that the host cell size increased at approximately 6 h postinfection (hpi) and the host nucleus enlarged at approximately 22 hpi, pointing to the importance of biochemical experiments. In addition, we showed that the intracellular structures could be quantitatively analyzed using multiple phase-contrast microscopy images with different focus positions at the same time point. Hence, morphological analyses of intracellular structures using phase-contrast microscopy, which have wide applications in live-cell observations, may be useful in studying various organisms that infect or are symbiotic with A. castellanii.

## INTRODUCTION

Acanthamoeba castellanii medusavirus (medusavirus), a member of the phylum *Nucleocytoviricota*, was isolated from hot spring water in Japan in 2019 (1). The medusavirus has a unique replication strategy of replicating the viral genome in the host nucleus without constructing visible viral factories in the host cytoplasm, such as those occurring in the families *Mimiviridae* and *Marseilleviridae* (1, 2). Transmission electron microscopy (TEM) has shown that the replication of the medusavirus genome does not degrade the host nucleus, unlike what is reported for pandoraviruses (2). The medusavirus genome contains homologous genes for Ran, DNA polymerase δ, and histones, which are proteins characteristic of eukaryotes. The ancestor of this virus is hypothesized to be related to the origin of the eukaryotic nucleus (2). In 2021, *Medusavirus stheno*, which was thought to be closely related to medusavirus (3), was isolated; thus, we are proposing a new genus, “Medusavirus,” and a new family, “*Mamonoviridae*,” in phylum *Nucleocytoviricota* (4). Acanthamoeba castellanii and related amoeba are considered to be the natural hosts of medusaviruses (1). The cytopathic effect (CPE) of the medusavirus causes rounding in infected A. castellanii cells at approximately 24 h postinfection (hpi) (5), even causing cyst formation in some cases (1). It has also been reported that this infection causes frequent rotational behavior and the formation of intercellular bridges in host cells (5). In A. castellanii and Acanthamoeba rhysodes, cells with abnormal nuclei, such as multinucleated cells, frequently form intercellular bridges (6, 7).

*Acanthamoeba* is an abundant eukaryotic microorganism that causes *Acanthamoeba*-associated keratitis (8). In the laboratory, this amoeba adheres to the bottom of a culture flask; it can change the shape of its cells and move around freely. Morphological and behavioral changes in living amoeba cells have been observed using phase-contrast microscopy. Image analysis of phase-contrast microscopy time-lapse movies of this amoeba has been described when the organisms are placed on a glass surface to investigate how to prevent keratitis (9) or under conditions of infection with giant viruses (5, 10–12). This amoeba is also known as a laboratory host for various giant viruses other than the medusavirus, including viruses from the families *Mimiviridae* (13), *Marseilleviridae* (14), and pandoraviruses (15). Previous studies have quantitatively revealed the morphological and behavioral changes in the contours of host A. castellanii cells caused by the CPE of eight giant viruses: three viruses from the family *Mimiviridae* (5, 10), three viruses from the family *Marseilleviridae* (5, 11, 12), one virus from the proposed family “Pandoraviridae” (5), and one virus of the species medusavirus (5).

The morphological changes caused by the CPE of a giant virus can be observed in the contours of both the cell and the organelles inside it, such as the nucleus and vacuoles. For example, in A. castellanii cells in the late stage of pandoravirus infection, TEM showed that the nucleoli became paler and progressively vanished and the nuclear envelope became invaginated and totally disappeared (15). In addition, analyses of time series changes in the viral factory of Acanthamoeba polyphaga mimivirus using a differential interference contrast microscope (16) and time series analysis showed changes in the actin filaments of mimivirus-infected *A. polyphaga* by using a fluorescence microscope (17). Such studies of intracellular morphology have been suggested to be helpful in understanding viral replication cycles (16).

In this study, we analyzed changes in the intracellular morphology of A. castellanii cells using phase-contrast microscopy. Image analysis for phase-contrast microscopy has been used to analyze intracellular mobility by measuring changes in intensity (18); however, it has not been determined whether phase-contrast microscopy can be used to analyze intracellular morphology. Although phase-contrast microscopy is widely used to observe live cells (19), it is difficult to use it in image analysis because of the existence of artifacts such as halos and shade-offs (18, 19). When analyzing intracellular morphologies using phase-contrast microscopy time-lapse movies, controlling the focus position is an issue. Compared with the outside contours of cells, the structures inside a cell are easily hidden by halos and shade-offs. Even within a single frame, the most suitable focal position for the analysis of intracellular morphology may differ depending on the morphology of each cell. Here, we updated the phase-contrast-based kinetic analysis algorithm for amoebae (PKA3), an image analysis algorithm we previously developed (5, 11), to implement analyses for multiple images with different focus positions at the same time point. This algorithm has made it possible to efficiently analyze intracellular morphology by capturing multiple images with different focus positions during time-lapse photography. Here, we aimed to reveal morphological time series changes in the contours of the vacuoles and nuclei of A. castellanii cells infected with four types of giant viruses, including medusavirus.

## RESULTS

### Performance of updated PKA3.

**FIG 1:**
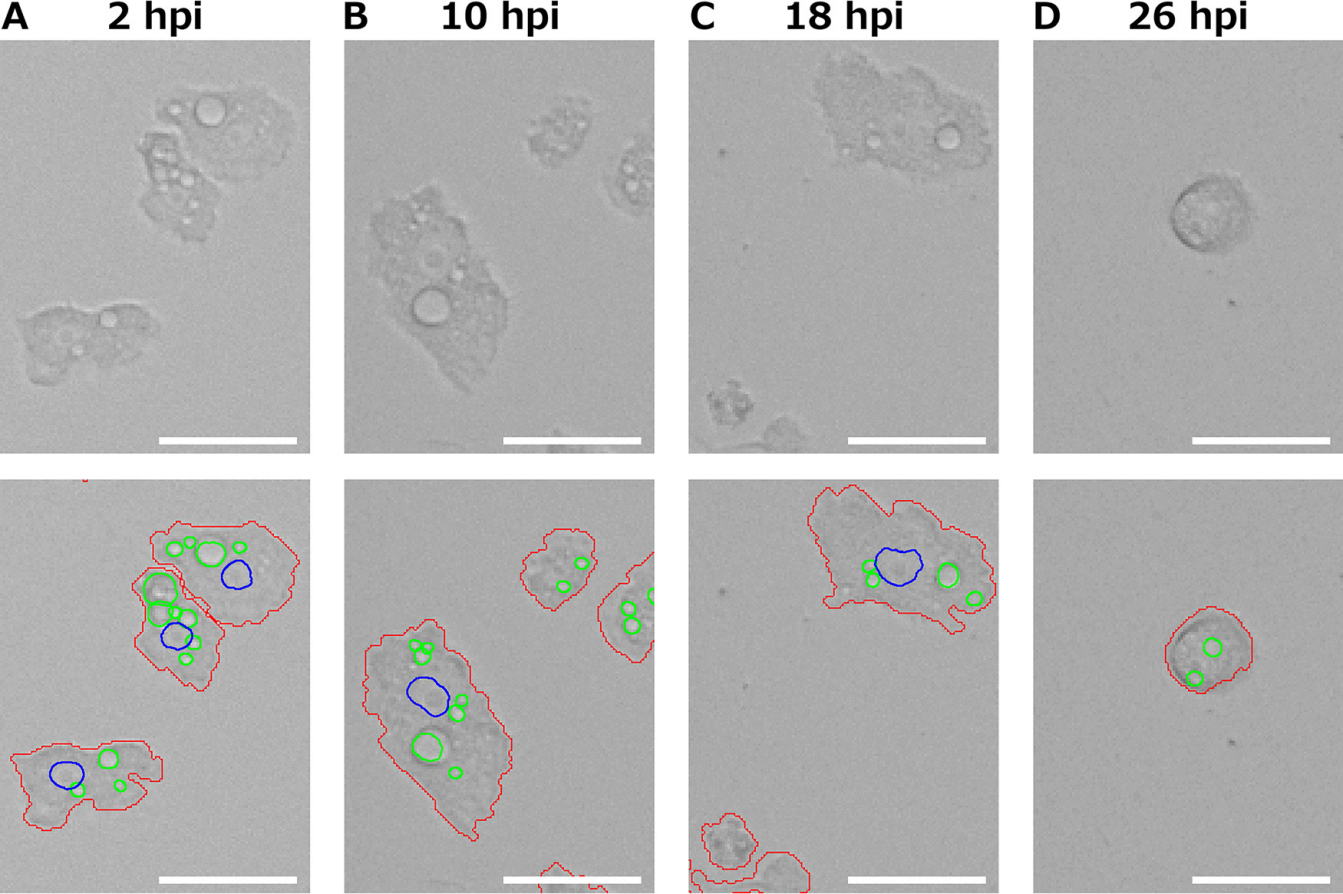
Contours of cells, nuclei, and vacuoles as detected by the PKA3 algorithm. Scale bar, 50 μm. The images in the upper panel are phase-contrast images of Acanthamoeba castellanii infected with medusavirus at 2 (A), 10 (B), 18 (C), and 26 hpi (D). The images in the lower panel are the corresponding PKA3 analysis results of the images in the upper panels. The red lines show the outlines of the particles considered to be cells, the blue lines show the outlines of the areas considered to be nuclei, and the green lines show the areas considered to be vacuoles.

**FIG 2:**
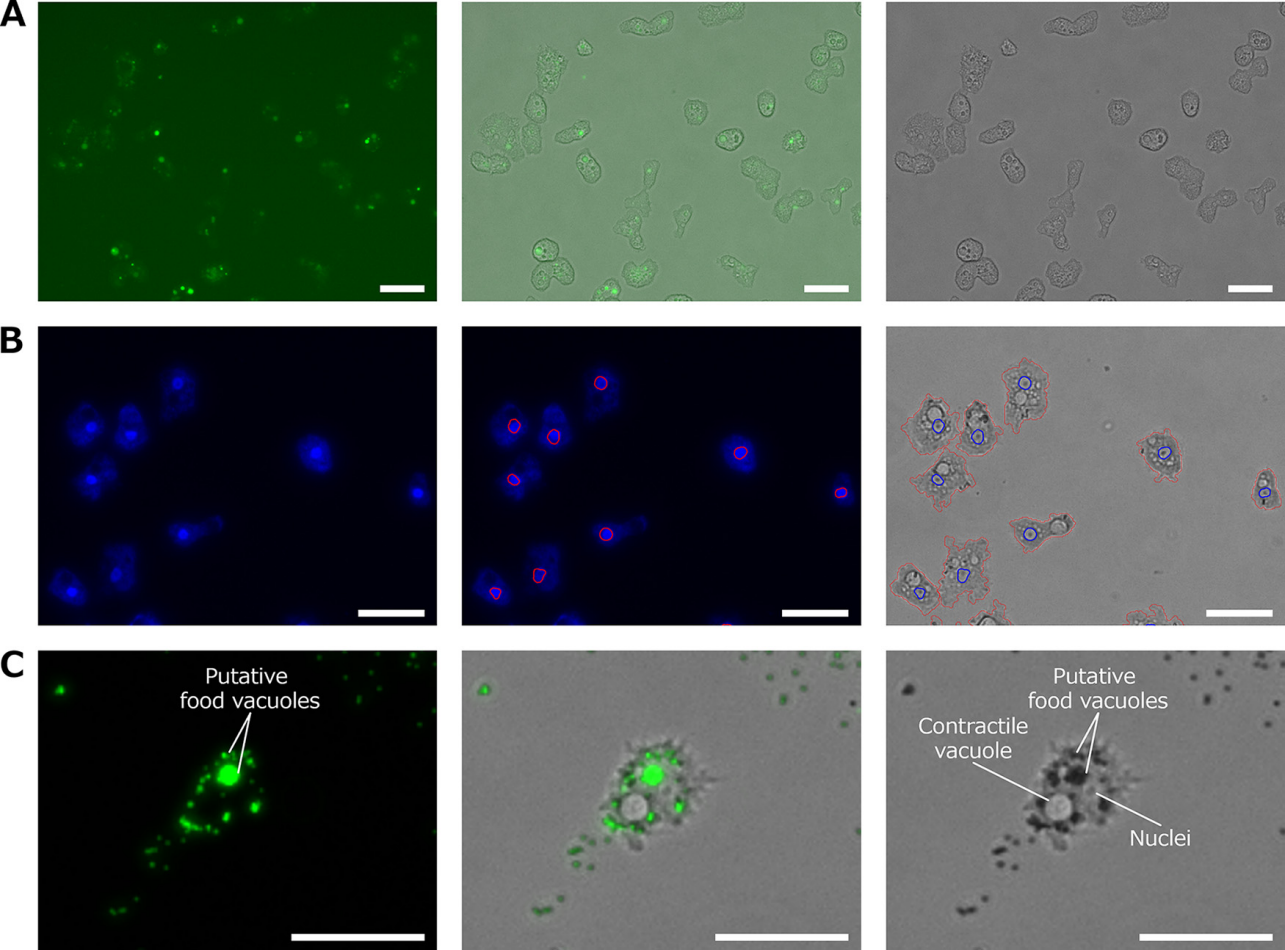
(A) A. castellanii infected with FITC-labeled medusavirus. (Left) Fluorescence microscopy; (right) phase-contrast microscopy; (middle) overlay image. (B) DAPI-stained A. castellanii. (Left) Fluorescence microscopy; (middle) fluorescence microscopy with analysis; (right) phase-contrast microscopy with analysis. Detected nuclei are indicated in red (middle) or blue (right), while detected cells are in red (right). (C) A. castellanii loaded with fluorescent latex beads. (Left) Fluorescence microscopy; (right) phase-contrast microscopy; (middle) overlay image. Scale bar, 50 μm.

Figure 1 shows the output images of medusavirus-infected A. castellanii analyzed using the updated PKA3 algorithm. In Fig. 1, the analyzed particles, the regions defined as cells by the PKA3 algorithm, are outlined in red. Nuclei and vacuoles detected using the PKA3 algorithm are outlined in blue and green, respectively. In the early stages of infection, when the outlines of the particles did not change, the nuclei were observed as double circles of phase intensity (blue line in Fig. 1A). In the late stage of infection, where the effects of CPE can be clearly observed, no nuclei were seen in the phase-contrast microscopy images, either visually or via analysis (Fig. 1D). Figure 2 shows fluorescence microscopy images for the infection of medusavirus and detection of nuclei and vacuoles. In Fig. 2A, the result of A. castellanii infected with fluorescein isothiocyanate isomer I (FITC)-labeled medusavirus is shown. FITC fluorescence (green in the left panel of Fig. 2A) was observed in all cells. Figure 2B shows the result of fixed A. castellanii stained with 4′,6-diamidino-2-phenylindole (DAPI). The overlay image of the nuclei detected using PKA3 algorithms (red line in middle panel of Fig. 2B) on fluorescence microscope images showed that the analysis results overlapped the nuclei shown via fluorescence (bright areas in left panel of Fig. 2B). Figure 2C shows the result of the uninfected A. castellanii loaded with fluorescent latex beads. Since the beads were phagocytosed (20, 21), food vacuoles show green fluorescence (left panel of Fig. 2C) and are black in the phase-contrast image (right panel of Fig. 2C). Beads were taken up by large and small vacuoles, except for the largest vacuole, which is thought to be a contractile vacuole.

**FIG 3:**
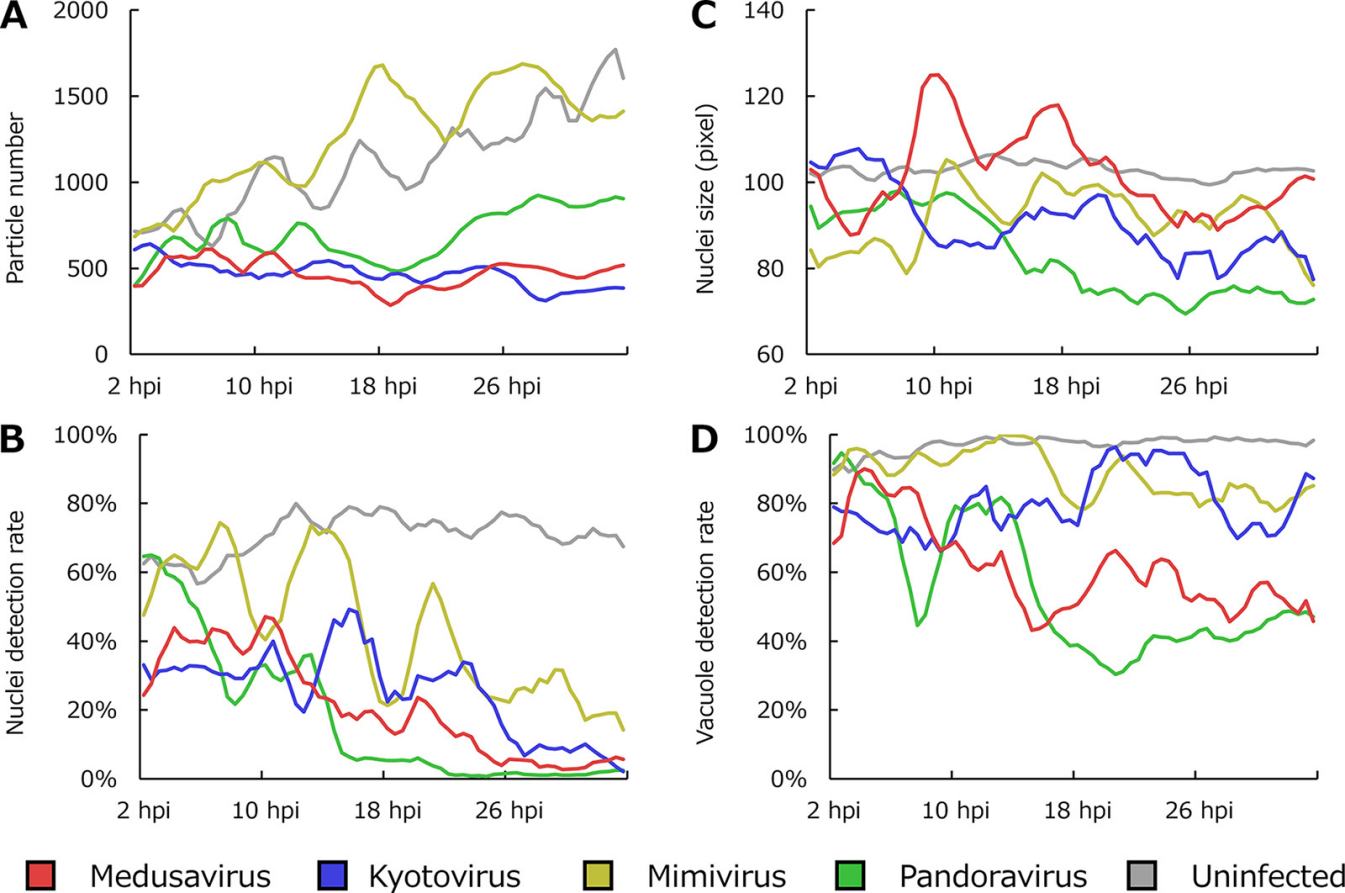
Analysis of A. castellanii cells infected with four types of viruses, namely, medusavirus, kyotovirus, *Mimivirus shirakomae*, and *Pandoravirus japonicus*. (A) Number of particles analyzed as cells. (B) Rate of cells with one nucleus detected. (C) Average sizes of the regions considered nuclei. (D) Rate of cells with one or more vacuoles detected. Each point in the line graphs represents the total (A) or average (B to D) values for particles obtained for 30 min (40 frames). Each series indicates a simple moving average for 4 data points. In panels B and D, the denominators were counted as 1 if the cell was tracked over multiple frames, while the numerators were counted as 1 if the intracellular structures were detected in one or more tracked frames.

Figure 3 shows the PKA3 algorithm analysis of A. castellanii infected with four viruses of phylum *Nucleocytoviricota*, including medusavirus, kyotovirus of the family *Marseilleviridae* (22), *Mimivirus shirakomae* of the family *Mimiviridae* (23), and *Pandoravirus japonicus* of the proposed family Pandoraviridae (24). Figure 3B shows the rate of A. castellanii cells with the nuclei detected in approximately 60 to 80% of uninfected cells but with a smaller rate of infected cells (Fig. 3B). In particular, the nuclei detection rate was <10% in pandoravirus-infected cells after approximately 16 hpi (green line in Fig. 3B). The average size of detected nuclei (Fig. 3C) indicated that the nucleus size of medusavirus-infected cells was larger than that of uninfected cells at some points (red line in Fig. 3C). Pandoravirus-infected cells had smaller nuclei than the other samples after approximately 16 hpi (green line in Fig. 3C), but the results were not statistically significant due to the limited data. Figure 3D shows the rate of A. castellanii cells containing detectable vacuoles. Kyotovirus- or mimivirus-infected cells maintained a >70% vacuole detection rate up to 34 hpi (blue or yellow line in Fig. 3D).

### Detection of cells and intracellular structures.

**FIG 4:**
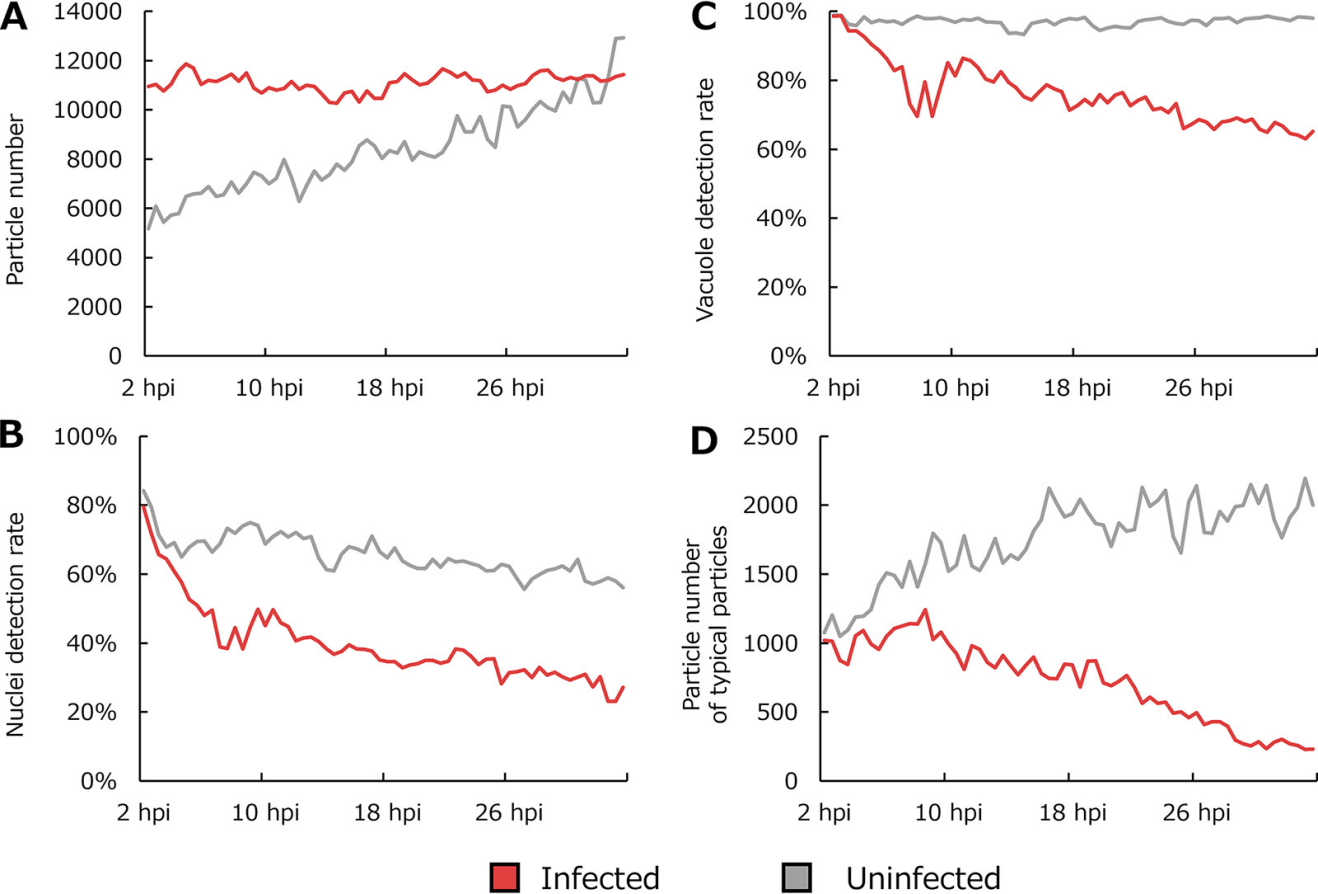
Analysis of the number of particles and detection rates of intracellular structures. (A) Number of particles analyzed as cells. (B) Rate of cells with one nucleus detected. (C) Rate of cells with one or more vacuoles detected. (D) Number of typical particles in panel A. Each point in the line graphs represents the total (A and D) or average (B and C) values for particles in all imaging data (10 data points for infected cells and 5 data for uninfected cells) obtained for 30 min (40 frames). In panels B and C, the denominators were counted as 1 if the cell was tracked over multiple frames, while the numerators were counted as 1 if intracellular structures were detected in one or more tracked frames.

**FIG 5:**
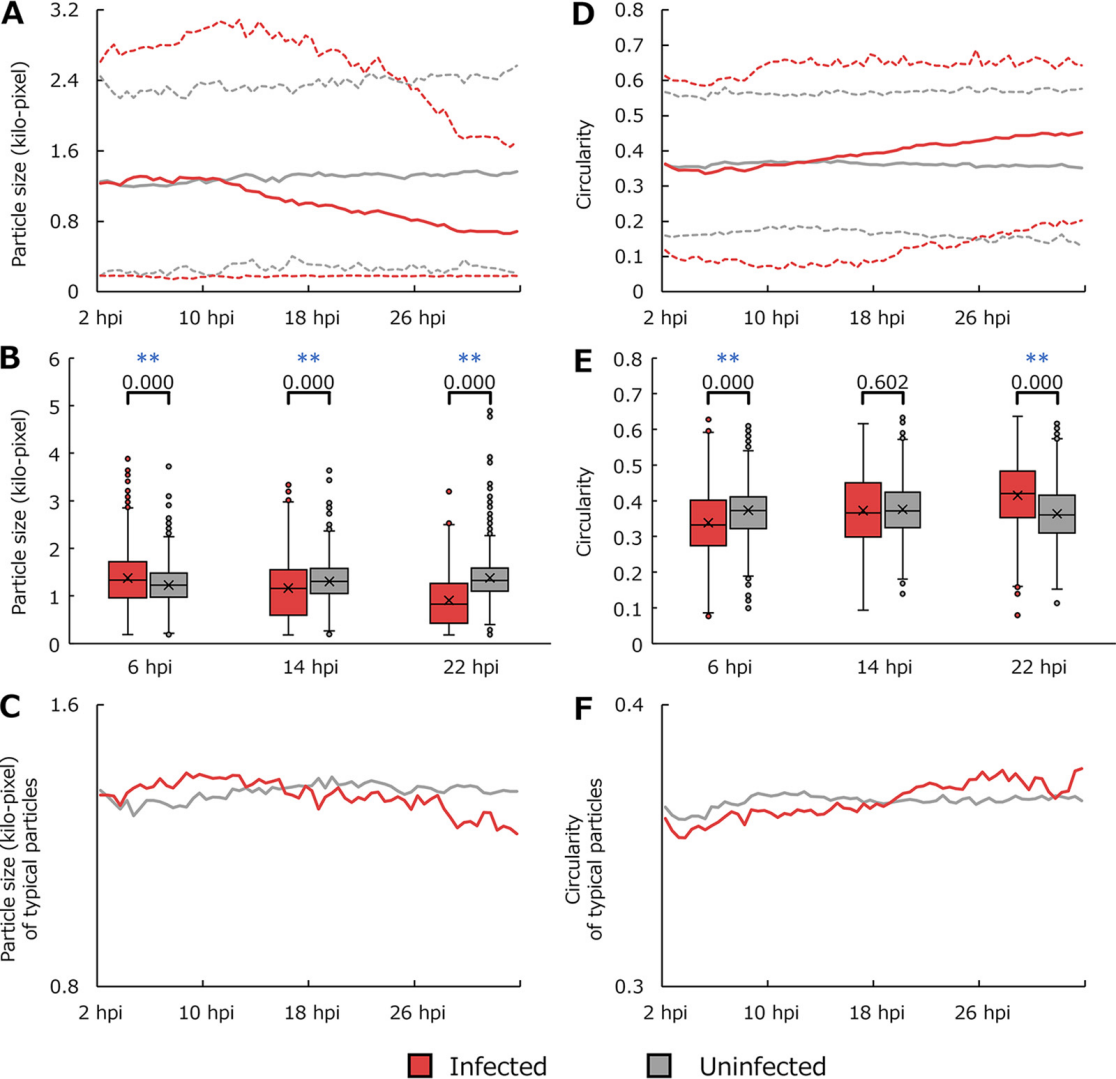
Analysis of size and circularity. (A) Average size of all detected particles. (B) Boxplot for data of panel A. (D) Average circularity of all detected particles. (C) Boxplot for data in panel D. The results shown in panels C and F are typical particle-only results for data shown in panels A and D, respectively. Each point in the line graphs is the average of values for particles in all imaging data (10 data points for infected cells and 5 for uninfected cells) obtained for 30 min (40 frames). The solid lines in panels A and D denote average values, while the dashed lines denote the maximum and minimum values, excluding outliers. Outliers were excluded using Tukey’s fences. The *P* values from the statistical analysis using the Mann-Whitney U test are shown in boxplots. **, *P* < 0.01.

**FIG 6:**
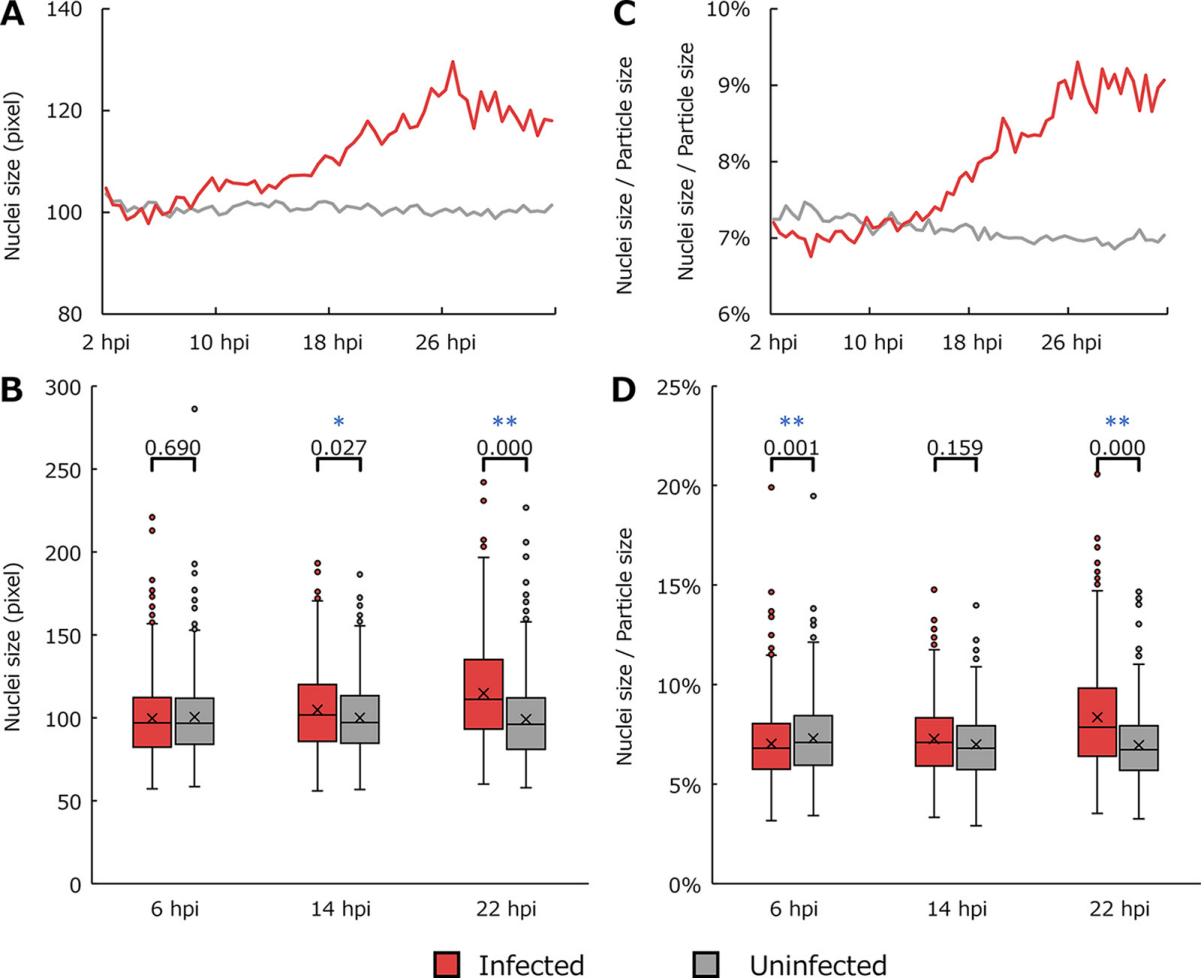
Analysis of nuclei. (A) Average sizes of the regions considered to be nuclei. (B) Boxplot for data shown in panel A. (C) Average ratios of the area of the nucleus to the area of the cells. (D) Boxplot for data shown in panel C. Each point in line graphs is the average of values for typical particles in all imaging data (10 data points for infected cells and 5 for uninfected cells) obtained for 30 min (40 frames). The *P* values from the statistical analysis using the Mann-Whitney U test are shown in boxplots. *, *P* < 0.05; **, *P* < 0.01.

**FIG 7:**
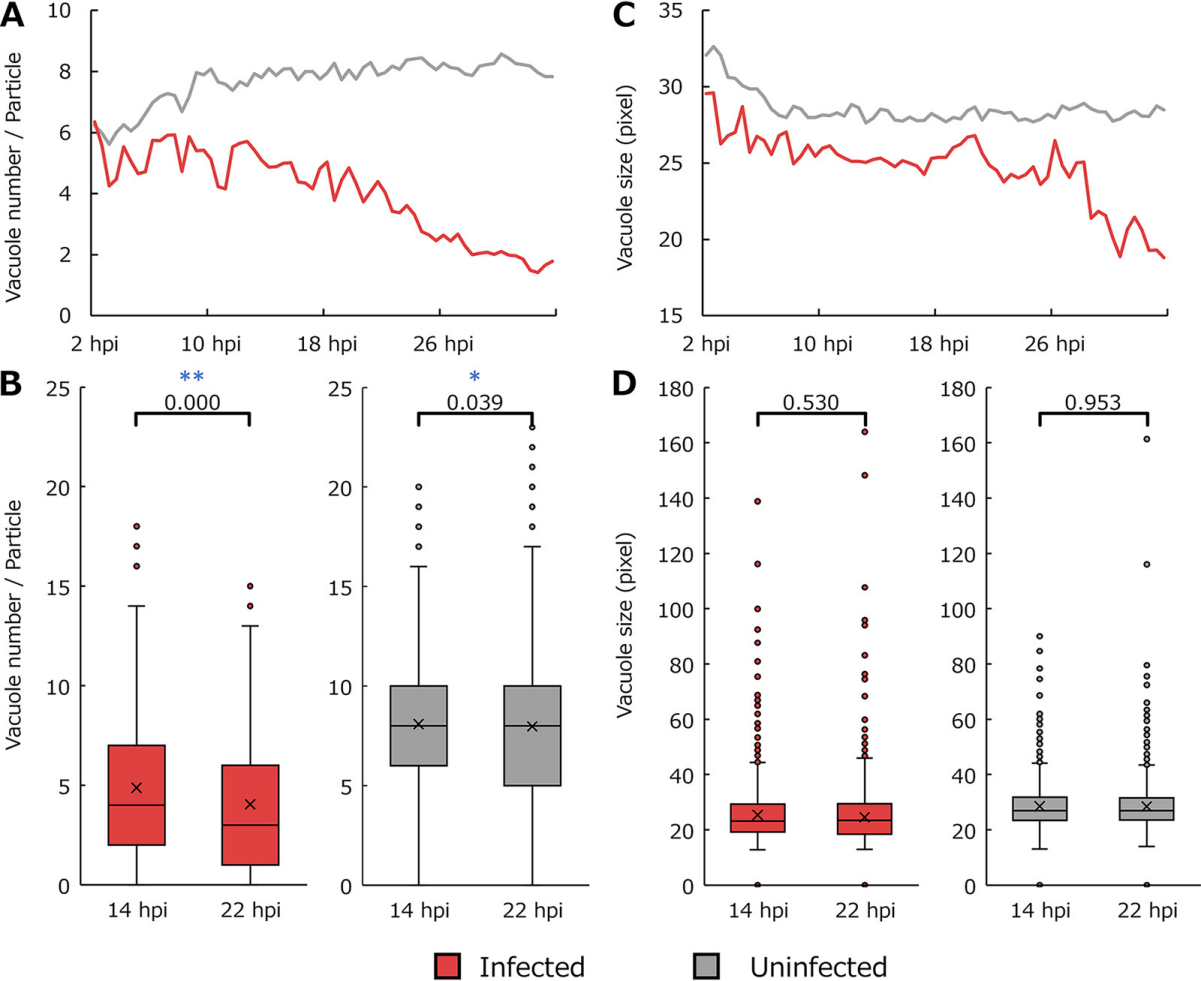
Analysis of vacuole. (A) Average number of regions considered vacuoles in each particle. (B) Boxplot for data shown in panel A. (C) Average sizes of regions considered vacuoles. (D) Boxplot for data shown in panel C. Each point in line graphs is the average value for typical particles in all imaging data (10 data points for infected cells and 5 for uninfected cells) obtained for 30 min (40 frames). The *P* values from the statistical analysis using the Mann-Whitney U test are shown in boxplots. *, *P* < 0.05; **, *P* < 0.01.

To reveal the intracellular morphological changes induced by medusavirus infection, phase-contrast time-lapse imaging data of 10 medusavirus-infected A. castellanii and five control A. castellanii cells were analyzed using the updated PKA3 algorithm (Fig. 4 to 7). (Here, “infection” refers to the infection of A. castellanii with medusavirus.) The medusavirus-infected sample used in Fig. 3 is not included in Fig. 4 to 7 due to differences in imaging conditions. Figure 4A shows that the number of detected particles was higher than that shown in Fig. 3A. The number of particles in uninfected cells gradually increased because of cell division, but the number of infected cells did not change (Fig. 4A). Nuclei were detected in approximately 60 to 80% of uninfected cells and 30 to 50% of infected cells (Fig. 4B). Vacuoles were detected in almost all uninfected cells and approximately 60 to 80% of infected cells (Fig. 4C).

As shown in Fig. 1D, the updated PKA3 algorithm could not detect the nuclei inside cells, which became rounded due to the CPE of medusavirus, thereby decreasing nuclei detection rate of infected cells. Visually identifying the nuclei of such cells is difficult; therefore, only particles with a typical A. castellanii cell morphology were used to analyze the nuclei and vacuoles.

Based on a previous study (5), a typical A. castellanii cell was defined as a particle with a size and circularity within 1 standard deviation from the mean of uninfected cells. Only particles with sizes of 902 to 1,979 pixels and circularities of 0.292 to 0.442 were used to analyze the contours of the nuclei. In this study, the detection of nuclei using the updated PKA3 algorithm was added to the requirements for cells with typical morphologies. The number of particles with typical A. castellanii cell morphologies (typical particle), among those detected via PKA3, is shown in Fig. 4D. The number of typical particles refers to the number of samples used in the average calculation and statistical analysis. The number of particles detected using the PKA3 algorithm in the infected samples did not decrease, since the CPE of medusavirus did not lyse host cells (red line in Fig. 4A). Meanwhile, the number of typical particles decreased due to changes in the morphology of host cells induced by the CPE of medusavirus (red line in Fig. 4D). Notably, after approximately 28 hpi, the number of samples used for statistical analysis was already small.

### Morphology of cell contours.

Figure 5 shows time series changes in the cells’ contours. The average particle size of the uninfected cells did not change, but that of the infected cells gradually decreased (solid line in Fig. 5A). The average circularity of the contours of the uninfected cells did not change, but that of the infected cells gradually increased (solid line in Fig. 5D). These results are consistent with previous results showing that the number of particles in medusavirus-infected cells did not increase and their contours became smaller and rounder over time (5). In addition, new results were obtained for previously unclear changes in particle size in the early stages of infection. The upper and lower dashed lines in Fig. 5A and D show the maximum and minimum particle sizes, respectively, excluding outliers. The maximum particle size indicated the appearance of large particles in the early stages of infection (dashed line in Fig. 5A). Up to approximately 10 hpi, the average particle size of infected cells was larger than that of uninfected cells (solid line in Fig. 5A). The minimum particle size of uninfected cells changed due to the presence of small healthy cells that could move out of the field of view (gray dashed line in Fig. 5A). On the other hand, the particles reduced in size owing to the CPE did not move and remained in the field of view; thus, the minimum particle size of infected cells did not change (red dashed line in Fig. 5A). Statistical analysis also indicated that the particle size was significantly different between infected and noninfected cells (Fig. 5B). The minimum circularity value of infected cells indicated the appearance of particles with more complex cellular contours than uninfected cells in the early stages of infection (dashed line in Fig. 5D). Statistical analysis also indicated that the circularity was significantly lower in the early stages of infection (Fig. 5E). These trends in the particle size and the circularity were observed not only in all particles but also in the typical particles (Fig. 5C and F).

### Morphology of nuclear contours.

Figure 6 shows time series changes in the nucleus of typical particles, i.e., particles whose size and circularity are within 1 standard deviation from the mean of healthy amoebae and whose nuclei are detectable. In contrast to the average size of particles, the average size of the nucleus gradually increased as the infection progressed (Fig. 6A). Statistical analysis indicated that the size of the nucleus was not significantly different between infected and noninfected cells at 6 hpi, but it was significantly larger in infected cells after 14 hpi, with the *P* value being smaller at 22 hpi than at 14 hpi (Fig. 6B). In infected cells, the ratio of the area of the nucleus to that of the particles was lower than that of uninfected cells before approximately 10 hpi, but gradually increased to exceed that of uninfected cells as the infection progressed (Fig. 6C). Statistical analysis also indicated that this ratio was significantly lower in infected cells than that in uninfected cells at 6 hpi. However, there was no significant difference at 14 hpi, but the number of infected cells was significantly higher at 22 hpi (Fig. 6D). Even though there were no significant differences in the size of the nuclei, the significantly lower nucleus area-to-cell area ratio in the early stages of infection was thought to reflect the larger size of the particles than that of the uninfected cells.

### Morphology of the vacuoles.

Figure 7 shows time series changes in the vacuoles of typical particles, i.e., particles whose size and circularity are within 1 standard deviation from the mean of healthy amoebae and whose nuclei are detectable. The number of vacuoles per particle gradually decreased after 10 hpi in infected cells (red line in Fig. 7A). Statistical analysis indicated that, in infected cells, the number of vacuoles per particle at 14 hpi was significantly lower than that at 22 hpi (red in Fig. 7B). There was also a significant difference in the number of vacuoles per particle between 14 and 22 hpi in uninfected cells; however, the *P* value for uninfected cells was larger than that for infected cells (gray in Fig. 7B). The average size of the vacuoles was generally lower in infected cells than in uninfected cells (Fig. 7C). However, infected cells appeared to have only slight changes in average vacuole size between approximately 10 and 26 hpi (Fig. 7C). Infected cells then showed a sharp decrease in the average vacuole size. Statistical analysis indicated no significant difference in the average size of vacuoles between 14 and 22 hpi in the infected cells (red in Fig. 7D). Similarly, no significant differences in the average vacuole size were observed in uninfected cells (gray in Fig. 7D).

## DISCUSSION

In this study, we used phase-contrast time-lapse imaging data to analyze changes in the intracellular morphology of A. castellanii infected with medusavirus. The analysis described in this report provides new insights into intracellular morphological changes caused by medusavirus infection. Up to approximately 12 hpi with medusavirus, the host A. castellanii cells became larger, whereas the size of the nucleus was largely maintained (Fig. 5A and 6A). After approximately 12 hpi, the cells became smaller and more rounded (Fig. 5), similar to the effects of CPE for many other giant viruses in *Acanthamoeba* cells (5). In addition, the results showed that under medusavirus infection, the nuclei of infected cells became larger and occupied a larger proportion of the cell area (Fig. 6). Furthermore, it was observed that the number of vacuoles in the infected cells decreased (Fig. 7A).

**FIG 8:**
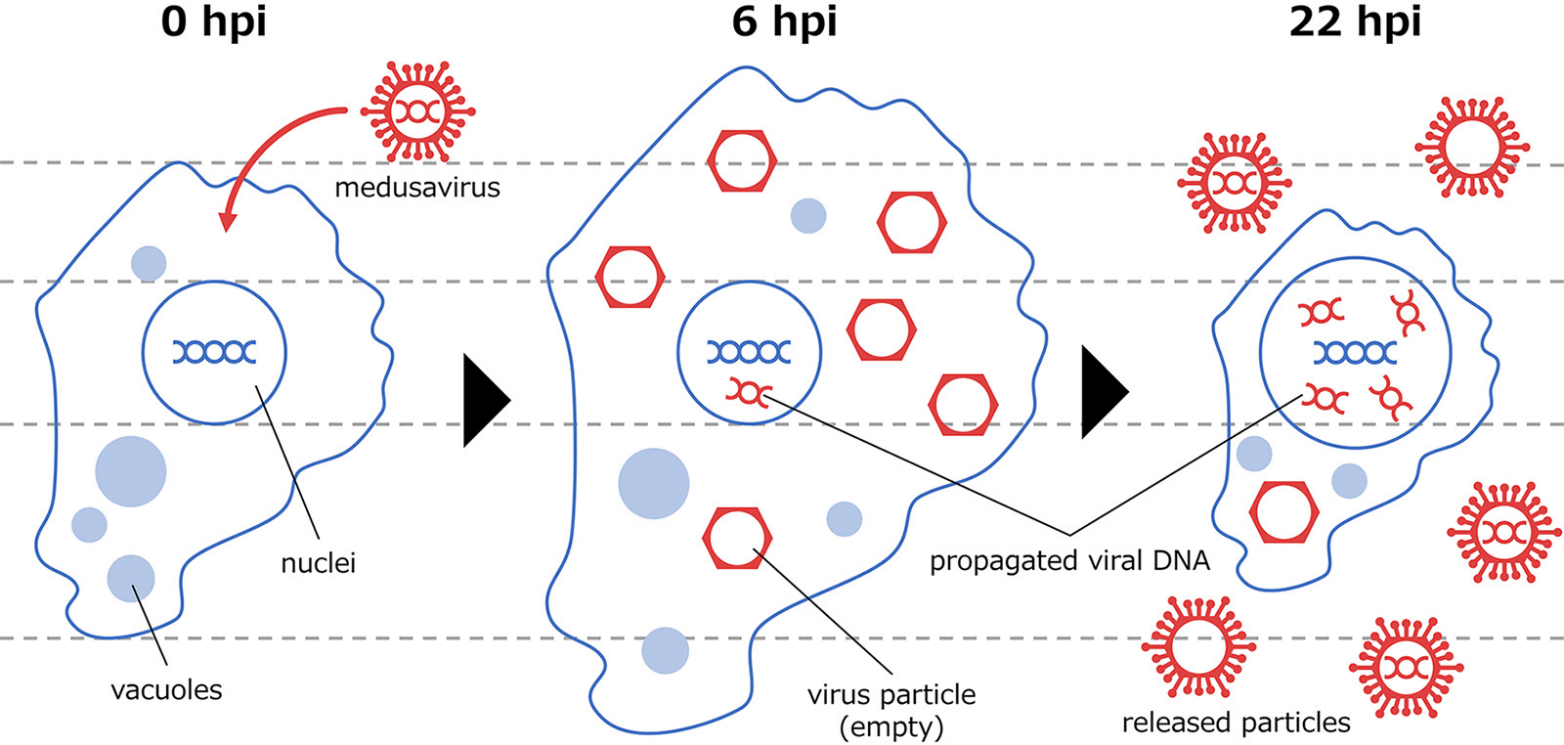
Hypothetical scenario describing the mechanism of medusavirus infection. At approximately 6 hpi, the presence of DNA-free viral particles in the host cytoplasm increases the size of the host cell. At approximately 22 hpi, the presence of replicated medusavirus DNA in the host nucleus increases the size of the host nucleus. Then, the size of the host cell is reduced because many medusavirus particles are released. It has been reported that some of the released medusavirus particles do not contain DNA (19).

Throughout the observation period, the number of particles, which are regions defined as amoeba by the PKA3 algorithm, did not increase in the medusavirus-infected samples (Fig. 4A). We also observed that most A. castellanii cells were invaded by medusavirus at 1 hpi (FITC-labeled virions) (Fig. 2A). Therefore, the ability of A. castellanii to divide was considered to be almost lost from the early stage of infection. One hypothesis that explains the mechanism underlying the emergence of larger cells before 12 hpi is that cells infected with medusavirus lost their ability to divide. Notably, the ratio of the size of the nucleus to that of the whole cell in infected samples was smaller than in uninfected samples (Fig. 6C). Another hypothesis explaining the emergence of larger cells in infected samples is that the presence of medusavirus particles that are not present in healthy amoeba cells increases the size of infected cells (Fig. 8). The formation of viral particles in the cytoplasm of A. castellanii cells has been reported in medusavirus infections (1). It has been observed using TEM that a large number of new medusavirus particles without DNA are present in the cytoplasm of cells 8 to 10 h after infection (1, 25). Additionally, medusavirus has been reported to replicate genomic DNA in the nucleus of their host A. castellanii cells (1). Furthermore, the transcriptional profiles of A. castellanii infected with medusavirus could reach the maximum proportion of viral mRNA in the total library at approximately 24 hpi (26). The results of this study showed that the average size of the nucleus reached a maximum at approximately 26 hpi, suggesting a relationship between nuclear enlargement and medusavirus genomic DNA replication (Fig. 6). One hypothesis explaining the host nucleus enlargement and cell size reduction is that the presence of medusavirus genomic DNA in the nuclei causes nuclear enlargement, while the release of mature viral particles from the cytoplasm reduces the cell size (Fig. 8). Additional investigations, such as inhibition of DNA replication, may help to clarify the cause of nuclear enlargement. In addition, as previously reported, the nucleus of the infected host did not degrade (2) and could still be detected and analyzed even at 32 hpi (Fig. 4A). Currently, there are still many unknowns in the relationship between medusavirus and the host nucleus, such as the mechanisms for replicating DNA in the host nucleus (26) and packaging DNA in viral particles (25). Time series analyses of the nuclear morphology of A. castellanii may be useful for understanding the viral propagation mechanism.

In this study, we analyzed the morphological changes in the vacuoles of A. castellanii cells during medusavirus infections. Most vacuoles of *Acanthamoeba* are food vacuoles involved in nutrient storage, digestion, and absorption or contractile vacuoles involved in water regulation (27). When an *Acanthamoeba* becomes a cyst, it excretes all food vacuoles (20). In our results, the decrease in the number of vacuoles (Fig. 7A) may be caused by the change in cell morphology due to the CPE of medusavirus infections. On the other hand, the decrease in the average size of vacuoles after 26 hpi (Fig. 7) was considered to be due to the disappearance or size reduction of contractile vacuoles of relatively large sizes, but no clear result was obtained because of the small number of typical particles observed. The food vacuoles of *Acanthamoeba* are also involved in the invasion mechanism of giant viruses. Although the invasion mechanism of the medusavirus has not been clarified, it has been reported that mimiviruses (16) and clandestinovirus, which have genomic and morphological properties similar to medusavirus (28), invade amoeba via phagocytosis. Since food vacuoles can be detected under a microscope using latex beads as in Fig. 2C and medusavirus particles can be detected using FITC labeling as in Fig. 2A or fluorescence *in situ* hybridization (1), a comparative analysis of their localization may be useful for understanding the mechanism of medusavirus invasion. The food vacuoles of *Acanthamoeba* were also of interest in a study of Legionella pneumophila, the bacterium responsible for legionellosis (29, 30). This bacterium is known to live in symbiosis with amoeba, including A. castellanii, and infects ciliates such as *Paramecium*. L. pneumophila invades amoeba via phagocytosis and evades the digestive system by altering the properties of the phagosome. Hence, biochemical analysis of the components contained in phagosomes may be useful for understanding the infection cycle of medusavirus.

**FIG 9:**
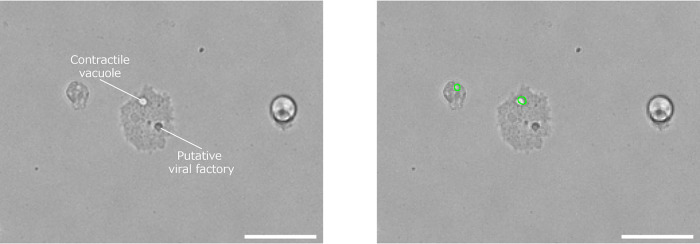
Phase-contrast microscopy images of A. castellanii infected with *Mimivirus shirakomae* at 24 hpi. The green lines in the right panel demonstrate the areas detected as vacuoles via the PKA3 algorithm. Scale bar, 50 μm.

A. castellanii infected with viruses of phylum *Nucleocytoviricota*, other than medusavirus, also showed new characteristics under phase-contrast microscopy. In pandoravirus-infected cells (green line in Fig. 3), the nuclear detection rate decreased after 16 hpi, possibly due to the disappearance of the nucleus of A. castellanii cells infected with pandoravirus (15). Since pandoravirus particles are large and can be seen under a phase-contrast microscope (5), it is also possible that the pandoravirus particles released from infected cells interfered with the analysis. In kyotovirus- or mimivirus-infected cells, vacuoles were detected in >70% of cells up to 34 hpi (blue or yellow line in Fig. 3D). The PKA3 algorithm does not distinguish between food and contractile vacuoles, unlike latex bead phagocytosis (Fig. 1 and 2C). A. castellanii cells infected with mimivirus showed contractile vacuoles until just before bursting; hence, the high vacuole detection rate was possibly due to contractile vacuoles (Fig. 9). Kyotovirus and mimivirus replicate in the host cytoplasm. In mimivirus-infected A. castellanii cells, structures derived from viral factories were observed (Fig. 9). In Fig. 9, contractile vacuoles were detected via the PKA3 algorithm, but viral factories were not. Viral intracellular structures may interfere with the analysis because of their similar appearance to the host nucleus. Also, identifying such structures may reveal the viral factory formation process of mimivirus. Therefore, additional imaging data for A. castellanii cells infected with viruses of phylum *Nucleocytoviricota* other than medusavirus may provide insights into intracellular structures. The limitation of this method is that the PKA3 algorithm cannot analyze the intracellular structure in cases where the nucleus disappears, such as in pandoravirus infection.

The image analysis in this study showed that phase-contrast microscopy images could be analyzed in more detail using images taken at multiple focus positions. The PKA3 algorithm allowed for the detection via phase-contrast microscopy of nuclei and vacuoles previously detected via fluorescence microscopy (Fig. 2). Phase-contrast microscopy can be used to observe living cells without any potentially damaging procedures, such as staining; therefore, it has a wide range of applications, such as the analysis of time series changes. In this study, we used this method to analyze changes in the nuclei and vacuoles of A. castellanii infected with medusavirus, but it could also be applied to changes caused by other viruses. However, the analysis results of the PKA3 algorithm are limited to morphological and behavioral changes in A. castellanii cells that are observable under phase-contrast microscopy. In addition, the PKA3 algorithm cannot analyze the intracellular structures that are altered in appearance. For example, the latex beads shown in Fig. 2C darken food vacuoles and hence cannot be analyzed using the algorithm without changing the settings and thresholds. While this can be an advantage in sensitively detecting changes in appearance, the algorithm cannot be used simultaneously with appearance-altering substances, which is a limitation. Biochemical experiments are required to further understand the timeline of viral infections and the relationship between the virus and its host. Our results may also aid in conducting optimal biochemical experiments to understand them. In addition to viruses, this analytical approach could also analyze changes in various organisms related to A. castellanii, such as L. pneumophila (29, 30), that interact with A. castellanii. Such results may help us understand various changes in A. castellanii, such as foreign particle invasion and symbiosis.

## MATERIALS AND METHODS

### Culturing A. castellanii cells.

Acanthamoeba castellanii (Douglas) Neff (ATCC 30010) cells were purchased from the American Type Culture Collection (ATCC; Manassas, VA, USA) and cultured in proteose-peptone-yeast extract-glucose (PYG) medium at 26°C according to the ATCC protocol as described in previous studies (1, 5, 22).

### Virus isolation and propagation.

Acanthamoeba castellanii medusavirus was isolated from water samples collected from Japanese hot springs. Viruses were isolated by screening A. castellanii cells for CPE in 96-well plates (1, 5). Each virus was propagated using A. castellanii cells in one or several 75-cm^2^ flasks and stored as a viral suspension in PYG supernatant at 4°C, as previously described (1, 5). Kyotovirus, *Mimivirus shirakomae*, and *Pandoravirus japonicus* were isolated and propagated as previously described (5).

### Medusavirus titration.

The multiplicity of infection (MOI) of each virus was calculated as previously described (5, 22). Briefly, 90 μL of PYG medium containing A. castellanii cells was added to each well of a 96-well microplate. Thereafter, 10 μL of PYG medium containing 10 serial 1:10 dilutions of the virus suspension was added to each well. The culture medium was incubated at 26°C for 4 days and then observed under a microscope. The virus titer was calculated using a 50% tissue culture infective dose (TCID_50_) calculator (Marco Binder, Department of Infectious Diseases, Molecular Virology, Heidelberg University; v2.1).

### Identification of intracellular structures in A. castellanii cells.

To identify medusavirus-invaded cells, infection of A. castellanii cells with FITC-labeled medusavirus was performed. FITC (Dojindo Laboratories, Kumamoto, Japan) dissolved in dimethyl sulfoxide was added to the medusaviruses collected in Na_2_CO_3_-NaHCO_3_ buffer solution at pH 9.0 to a final concentration of 50 ng/mL and mixed by inversion for 12 h. The FITC-labeled medusavirus was washed three times with phosphate-buffered saline (PBS) and added to PBS containing A. castellanii at a density of 20,000 cells/mL, cultured in 12-well plates, and incubated at 26°C for 1 h. After washing with PBS (three times) to remove free virions, phase-contrast and fluorescence microscopy images were then taken using an all-in-one fluorescence microscope (BZ-X800/X810; Keyence Co., Osaka, Japan).

To identify the nuclei, DAPI staining of A. castellanii cells was performed as previously described (1). Briefly, A. castellanii cells cultured in glass dishes were washed twice with PBS and fixed with methanol. After 20 min, the methanol was removed and the glass dishes were air dried. Cells were then stained with a solution containing 500 ng/mL DAPI in PBS for 5 min; the staining solution was removed and phase-contrast and fluorescence microscope images were taken using an all-in-one fluorescence microscope.

To identify the vacuoles, uptake of fluorescent latex beads into A. castellanii cells was performed as previously reported (20, 21). Briefly, PBS containing A. castellanii at a density of 20,000 cells/mL cultured in a shaker (DSR-2200; Kenis Ltd., Osaka, Japan) for 1 h was prepared in a 12-well plate. Beads (1.0-μm carboxylate-modified polystyrene, fluorescent yellow-green; Sigma-Aldrich Japan G.K., Tokyo, Japan) were added to cells to a final concentration of 300 ng/mL and incubated at 26°C for 2 h. After washing with PBS (three times), phase-contrast and fluorescence microscopic images were taken using an all-in-one fluorescence microscope.

### Obtaining time-lapse images.

Time-lapse phase-contrast imaging data for A. castellanii cells were obtained using an all-in-one fluorescence microscope with a 20× lens objective (CFI Plan Fluor DL 20×; Nikon Instech, Tokyo, Japan). This microscope allows time-lapse microscopic imaging in bright-field, phase-contrast, and fluorescence modes. In addition, images at multiple focal positions within the same time point can be obtained. A Precision 3430 desktop computer (Dell Inc., Round Rock, TX, USA) with a CPU (Xeon E-2124; Intel Co., Santa Clara, CA, USA), 16.0 GB LPDDR4x RAM, and GPU (Quadro P400; Nvidia Co., Santa Clara, CA, USA) was used to operate the microscope.

For time-lapse imaging, PYG medium containing A. castellanii at a density of 40,000 cells/3 mL and medusavirus at an MOI of 100 was prepared. The number of amoeba cells was determined using a disposable cell counter (WC2-100; WakenBtech Co., Kyoto, Japan). PYG medium (150 μL) was added to each well of a 96-well microplate and diluted with distilled water to a total volume of 400 μL, resulting in an A. castellanii suspension with a concentration of 5,000 cells/mL. The sample was left under the microscope for 2 h until the amoeba adhered to the bottom of the well, and then the cells were imaged in time-lapse mode for 32 h. As a control, PYG medium containing A. castellanii at a density of 40,000 cells/3 mL without medusavirus was prepared. Time-lapse imaging of the control samples was performed using the same procedures as those for the infected samples. Time-lapse imaging was performed 10 times on infected samples prepared with different A. castellanii cultures and five times on control samples prepared with different A. castellanii cultures. The time-lapse imaging data consisted of multiple 960- by 720-pixel images with 256 grayscale tones. The imaging data contained 2,560 frames, taken every 45 s for 32 h, with each frame containing 16 images at different focus positions at a 1.2-nm pitch, consisting of a total of 40,960 images.

The type-lapse imaging data for comparing the four types of viruses, namely, medusavirus, kyotovirus, *Mimivirus shirakomae*, and *Pandoravirus japonicus*, were the same as previously reported (5). The amoebae, MOI, and microscope used were the same as the imaging data above. Unlike the imaging data above, cells were cultured in a 12-well microplate, the amoeba cell concentration was 20,000 cells/mL, and each frame contained 8 images with different focus positions.

### Updating the PKA3 algorithm.

To analyze the intracellular morphology from phase-contrast microscope images, we updated the PKA3 algorithm (5, 11), which was written using the C++ language. The new algorithm used all 16 images at different focus positions. Different focus positions in phase-contrast microscopy result in different phase intensities for each image pixel. For pixels at the coordinates where the phase-shifting object is present, the phase intensity changes significantly with respect to the focus position. The difference in that change depends on the intensity of the phase shift. The effect of artifacts was also changed with respect to the focus position. The new algorithm created a two-dimensional (2D) map by linearly approximating and parameterizing the differences in phase intensity with respect to the focus position for each pixel. This 2D map was analyzed using the PKA3 algorithm as previously reported (5, 11). Briefly, the difference in intensity between each pixel in the 2D map and its neighbors was used to detect particles while avoiding phase-contrast microscopy artifacts.

The outputs of the algorithm include the number of particles in the frame and the size, circularity, and elongatedness of each particle, as previously reported (5). Circularity was calculated using the equation 4πS/L^2^, where S is the area of the particle and L is the perimeter of the particle (31). Elongatedness was obtained by dividing the length of the minimum bounding rectangle at the widest angle by the width of the minimum bounding rectangle at the narrowest angle (32). Because PKA3 performs particle tracking, it is possible to obtain the moving distance and diffusion coefficient (33) of the particles, but this was not used in this study. However, tracking also has a slight effect on the morphological results, as the tracking results are used to prevent the incorrect cutting and binding of particles.

In addition, the proposed algorithm could analyze the internal structure of a particle. Because the nuclei and vacuoles (the targets of the analysis) are both circular, the algorithm detects circular structures within a particle. To do this, the algorithm first detects imperfect circles centered on specific pixels, which are within the particle and have an intensity of the two-dimensional extremum. The region of each circle is defined as the region closest to the perfect circle when the region is expanded such that the difference in intensity from the center pixel gradually increases. The algorithm then calculates the circularities, average radii, and intensity differences of the detected circles. Finally, the algorithm defines the best double circle with a circularity of at least 0.85 as the cell nucleus. Among the other circles, those with a circularity of at least 0.95 and a radius of at least 2 pixels were defined as vacuoles. The algorithm then calculates the size and circularity of each nucleus and the number and average size of vacuoles in each particle.

A Microsoft Surface Laptop 4 computer (Microsoft Co., Redmond, WA, USA) with an i7-1185G7 CPU (Intel Co.), 32.0 GB of memory, and an Intel Iris Xe Graphics GPU was used to develop the program and analyze the images. All images in the time-lapse imaging data were stored in a built-in solid-state drive. With this setup, an average of approximately 36 min was required to analyze the imaging data generated in this study.

### Statistical analysis.

The statistical significance of each result was analyzed using the two-sided Mann-Whitney U test (Fig. 5B and E, Fig. 6B and D, and Fig. 7B and D). The *n* and *P* values of the Mann-Whitney U test are shown in each figure. Microsoft Excel was used to create graphs and calculate the standard deviations.

### Data availability.

The microscopy images taken in this study, the source code of the PKA3 algorithm, and the file outputs from the analyses using PKA3 are available at https://pkaaa2022.z11.web.core.windows.net.
